# Socioeconomic disparities and its association with food security, diet quality, and growth among Malaysian children aged 6 months to 12.9 years

**DOI:** 10.3389/fpubh.2025.1709696

**Published:** 2025-12-24

**Authors:** Pei Teng Lum, Jasmine Siew Min Chia, Giin Shang Yeo, See Meng Lim, Mohd Jamil Sameeha, Jyh Eiin Wong, Nik Shanita Safii, Ilse Khouw, Bee Koon Poh

**Affiliations:** 1 Faculty of Health Sciences, Centre for Community Health Studies (ReaCH), Universiti Kebangsaan Malaysia, Kuala Lumpur, Malaysia; 2 School of Pharmacy, Monash University Malaysia, Subang Jaya, Malaysia; 3 Obesity-UKM Research Group, Universiti Kebangsaan Malaysia, Bangi, Malaysia; 4 FrieslandCampina, Amersfoort, Netherlands

**Keywords:** child hunger, diet quality, food security, growth, socioeconomic disparities

## Abstract

**Introduction:**

Malnutrition and poor growth among children remain a public health challenge, largely driven by socioeconomic disparities that limit access to nutritious food. This study examines association of socioeconomic characteristics with food security, diet quality, and growth among Malaysian children.

**Methods:**

Cross-sectional data from South East Asian Nutrition Surveys (SEANUTS II) Malaysia, involving 2,973 children aged 0.5–12.9 years, were analysed. Socioeconomic characteristics, including information on children and households, were collected via questionnaires. Food insecurity was assessed using the 10-item Radimer/Cornell instrument. Dietary intake was assessed using a 24-h recall, with diet quality determined by Mean Adequacy Ratio (MAR), derived from average Nutrient Adequacy Ratio for 15 nutrients based on Estimated Average Requirement (EAR). Growth outcomes were examined using body mass index-for-age (BAZ), height-for-age (HAZ), and weight-for-age (WAZ) z-scores. Complex samples ANOVA compared food security, diet quality, and growth indicators with socioeconomic characteristics.

**Results:**

Over half of the children (59.8%) were from low-income households. Although 47.8% had low food expenditure and 41.7% were food insecure, overall diet quality was reflected in MAR scores of 0.84 (maximum = 1.00). Child hunger was more common in rural areas, in households with lower parental education, unemployed mothers, lower income, lower food expenditure, and more siblings (*p* < 0.01). Younger, urban children, boys, Chinese or other ethnicities, from households with higher maternal education, and fewer siblings had better diet quality (*p* < 0.05). Lower HAZ and WAZ were more common among younger children, Malays, households with non-working mothers, lower incomes, lower food expenditures, and more siblings (*p* < 0.05). Children from rural areas had lower HAZ (*p* < 0.01). Younger children, girls, lower household income, and lower food expenditure had lower BAZ (*p* < 0.05).

**Conclusion:**

Socioeconomic disparities are significantly associated with compromised food security, poorer diet quality, and suboptimal growth among Malaysian children. Findings highlight the importance of addressing socioeconomic inequalities through targeted policies and evidence-based interventions ensuring equitable nutritional health and growth among vulnerable children.

## 1 Introduction

Optimal nutrition during childhood is fundamental not only for physical growth, but also for cognitive development, immune competence, and the prevention of adverse long-term health outcomes (1). However, the ability to attain adequate dietary intake is often compromised by socioeconomic constraints, as evidenced in many low- and middle-income countries, including Malaysia, as demonstrated in a systematic review by Sulaiman et al. (2).

Socioeconomic disparities, such as low parental education, limited household income, unemployment and large family size, can significantly limit access to nutritious food and healthcare (3). These inequalities have been exacerbated by the COVID-19 pandemic, as disadvantaged households are often dependent on low-paid, unstable jobs, and experienced prolonged disruptions to their livelihoods. Many of these labour-based activities were discontinued during lockdowns, resulting in widespread job loss and exacerbating income inequality, particularly among families with low- and middle-income (4). As a result, children from disadvantaged families were at increased risk of undernutrition, compromising their growth and development (5). Besides, the pandemic also intensified food insecurity, particularly in larger households, highlighting the barriers to adequate nutrition (6). In particular, the role of mothers should be emphasised, as their education and employment status have been consistently identified as key determinants of household food security and child nutritional outcomes (7).

In Southeast Asia, children experience double burden of malnutrition, characterised by the co-existence of undernutrition and overnutrition. Regional data from the SEANUTS indicate that stunting and underweight remain prevalent, especially among children with low dairy consumption (8), while the prevalence of overweight and obesity is increasing in urbanised settings (9). In Malaysia, this transition is even more pronounced as highlighted in national surveys. SEANUTS reported notable rates of stunting among children below 4 years, while overweight and obesity remain prevalent, particularly among primary school-aged children (10). Furthermore, National Health and Morbidity Survey (NHMS) 2024 confirmed a continuing double burden of malnutrition, with stunting (8.1%), overweight (14.4%), and obesity (13.6%) persisting among children and adolescents (11). Many Malaysian children fail to meet the recommended intakes for calcium and vitamin D, and irregular meal patterns, fast food consumption, and low fruit and vegetable intake are common (12–14). Socioeconomic and regional differences are also apparent, with rural children exhibiting higher rates of undernutrition, whereas urban children show greater risks of overnutrition and lower calcium intake (15).

Despite the progress Malaysia has made in national nutrition programmes, socioeconomic inequalities persist, affecting access to food and health resources, particularly among children (16). While previous national studies (17–20) have explored malnutrition trends, limited recent data are available on how specific socioeconomic factors shape food security, diet quality, and growth outcomes collectively in Malaysian children.

Therefore, this study aims to address this gap by examining the associations between socioeconomic indicators and three critical domains: food security, diet quality, and growth, using data from the SEANUTS II study in Malaysia. Understanding these relationships is crucial for designing equitable, evidence-based nutrition policies and interventions that prioritise vulnerable groups.

## 2 Methodology

### 2.1 Study design

Data from SEANUTS II Malaysia, which was collected between May 2019 and March 2020, was used for this analysis. A total of 2,989 children aged 0.5 to 12.9 years were recruited through a multistage cluster sampling approach across four regions of Peninsular Malaysia: Central, Northern, East Coast, and Southern. The initial stage of sampling involved selection by the Department of Statistics Malaysia (DOSM), where one rural and one urban area were selected from different states to represent the population of each region. In the second stage, DOSM provided a list of enumeration blocks (EB) within these areas. All households and educational institutions located within a 5 km radius of the selected EB were considered eligible for participation in this study.

The design and protocols of the study involving human subjects have been described in detail in previous published work (10, 21). All procedures were conducted in accordance with the principles of the Declaration of Helsinki, approved by the Research Ethics Committee of Universiti Kebangsaan Malaysia (JEP-2018-569), and registered with the Dutch Trial Registry (NL7975). All necessary permissions, including parental consent and child assent, were obtained prior to data collection.

### 2.2 Socioeconomic characteristics and household food security status

Parents/guardians completed questionnaires on socioeconomic characteristic information (age, sex, area of residence, ethnicity, region, number of siblings, parental education, maternal employment status, and monthly household income and food expenditure). Monthly household food expenditure was classified relative to the national mean [MYR 783; 1 US Dollar = MYR 4.13 (as of November 14, 2025)], for food and non-alcoholic beverages, as reported in the Household Expenditure Survey Report 2019 (22). Food security status was assessed using a validated 10-item Radimer/Cornell Hunger and Food Security instrument (23), which evaluates the household food security condition with three response options: not true (negative), scored as “0”; sometimes true, or always true (positive), scored as “1” and “2,” respectively. Responses were subsequently scored and classified into four levels of increasing severity: household food secure (negative responses to all items), household food insecure (positive responses to any of the first four items), individual food insecure (positive responses to any of the next three items), and child hunger (positive responses to any of the last three items).

### 2.3 Dietary intake and diet quality

A one-day, triple-pass 24 h recall approach (24) was used to assess dietary intake. Interviews were conducted either by phone or face-to face with parents/guardians/caregivers for children aged 0.5 to 9 years, and with children aged 10 to 12 years themselves. Participants were shown household measurement booklets (for phone interviews) or standard household utensils, such as bowls, cups, spoons, and ladles, to facilitate portion size estimation. Nutrient analysis was conducted using NutritionistPro software (Axxya Systems LLC, Redmond, Washington, United States), with the Malaysian Food Composition Database (25) serving as the primary reference. Implausible dietary reports were excluded based on assessed energy intake to predicted energy expenditure ratio using the method suggested by Black and Cole (26), with an acceptable range of 0.27–1.73. Diet quality was evaluated using Mean Adequacy Ratio (MAR) (27), calculated by averaging Nutrient Adequacy Ratio (NAR, truncated at 1.0) of 15 nutrients, including energy, carbohydrate, protein, fat, vitamin A, vitamin C, thiamine, riboflavin, cobalamin, vitamin D, phosphorus, calcium, iron, potassium, and sodium. Each NAR was derived by dividing the children’s nutrient intake by the respective Estimated Average Requirement (EAR) or Adequate Intake (AI) values from the Malaysian Recommended Nutrient Intakes (RNI) 2017 (28). For macronutrients (carbohydrates, protein, and fat), NARs were expressed in the percentage contribution of each macronutrient to total energy intake, and compared against the acceptable macronutrient distribution range (AMDR). Higher MAR scores indicated better overall diet quality.

### 2.4 Anthropometric measurements

Body weight was measured using digital scales: SECA 354 (SECA GmBH, Germany; precision: 0.01 kg) for infants and SECA 874 (SECA GmBH, Germany; precision: 0.05 kg) for older children. For children aged under 2 years, recumbent length was measured using either SECA 210 measuring mat or SECA 417 infantometer (SECA GmBH, Germany; precision: 0.1 cm). Standing height for older children was measured using SECA 213 stadiometer (SECA GmBH, Germany; precision: 0.1 cm).

Anthropometric z-scores, including height-for-age (HAZ), weight-for-age (WAZ) and body mass index-for-age (BAZ), were calculated using WHO software (World Health Organization, Geneva, Switzerland): Anthro version 3.2.2. for children aged under 5 years (29) and AnthroPlus version 1.0.4 for children aged ≥ 5 years (30). Nutritional status was classified following WHO Growth Standards for children aged 0 to 4.9 years (31), and WHO Growth Reference for those aged 5 to 19 years (32). As also reported by Poh et al. (10), children with implausible z-scores (*n* = 16), defined using WHO-recommended cut-offs (HAZ and WHZ < −6 or > + 6; WAZ and BAZ < −5 or > + 5), were excluded from further analysis.

### 2.5 Statistical analysis

Statistical analyses were performed using the Complex Samples module in IBM SPSS Statistics for Windows version 22.0 (IBM Corp, Armonk, New York, United States). Weight factors were derived based on the projected Malaysian population aged 0.5–12 years in 2019, using data from the 2010 Malaysian census database (33). Descriptive statistics were expressed in percentages, 95% confidence interval, means, and standard errors. Pearson Chi-square test compared non-child hunger and child hunger groups across categorical socioeconomic characteristics. One-way ANOVA tests compared continuous variables of growth indicators and diet quality. Statistical significance was set at *p* < 0.05 (two-tailed).

## 3 Results

A total of 2,973 children, representing an estimated population of 4,904,279 Malaysian children aged 0.5 to 12.9 years, were included in this study. Table 1 shows the socioeconomic characteristics and food security status of the children’s household. The majority (59.8%) belonged to low-income households (B40 group). Nearly half of the parents/guardians (47.8%) reported low monthly household food expenditure (< MYR 783). Overall, 41.7% of households experienced some form of food insecurity, comprising 18.2% with household insecurity, 6.3% with individual insecurity, and 17.2% with child hunger.

**Table 1:** Sociodemographic characteristics and household food security among Malaysian children (*n* = 2,973).

Characteristics	Unweighted count (*n*)	Estimated population	Percentage (%)	95% CI	Mean	SE
All	2,973	4,904,279				
Age (years)					6.7	0.1
Age group						
0.5–0.9 years	76	191,818	3.9	3.0–5.1		
1.0–3.9 years	555	1,172,103	23.9	21.9–26.0		
4.0–6.9 years	919	1,201,761	24.5	22.8–26.3		
7.0–12.9 years	1,423	2,338,597	47.7	45.4–49.9		
Sex						
Boys	1,418	2,526,203	51.5	49.3–53.7		
Girls	1,555	2,378,076	48.5	46.3–50.7		
Residence						
Rural	881	1,304,152	26.6	24.7–28.5		
Urban	2,092	3,600,127	73.4	71.5–75.3		
Ethnicity						
Malay	1,782	3,651,991	74.5	72.6–76.2		
Chinese	867	773,145	15.7	14.6–17.0		
Indian	279	304,226	6.2	5.4–7.1		
Others	45	174,917	3.6	2.4–5.2		
Region						
Central	672	1,429,940	29.2	26.9–31.5		
East coast	782	1,264,697	25.8	24.0–27.7		
Northern	772	1,067,972	21.7	20.2–23.5		
Southern	747	1,141,670	23.3	21.6–25.0		
Paternal education	2,818	4,620,668				
Non-schooling/Primary school	140	197,325	4.3	3.4–5.3		
Secondary school	1,489	2,425,298	52.5	50.2–54.7		
Tertiary school	1,189	1,998,045	43.2	41.0–45.5		
Maternal education	2,943	4,854,907				
Non-schooling/primary school	116	171,624	3.5	2.8–4.5		
Secondary school	1,391	2,287,623	47.1	44.9–49.3		
Tertiary school	1,436	2,395,660	49.4	47.1–51.6		
Paternal employment status	2,823	4,628,387				
Not working	74	131,185	2.8	2.1–3.8		
Working	2,749	4,497,202	97.2	96.2–97.9		
Maternal employment status	2,933	4,840,139				
Not working	1,073	1,815,695	37.5	35.4–39.7		
Working	1,860	3,024,444	62.5	60.3–64.6		
Total monthly household income^a^	2,916	4,824,481			5,018	102
B40 (< MYR 4850)	1,683	2,884,627	59.8	57.6–62.0		
M40 (MYR 4850–10,959)	995	1,538,247	31.9	29.9–34.0		
T20 (> MYR 10959)	238	401,607	8.3	7.1–9.7		
Monthly household food expenditure^b^	2,895	4,780,285			1,075	22
< MYR 783	1,217	2,286,899	47.8	45.6–50.1		
≥MYR 783	1,678	2,493,386	52.2	49.9–54.4		
Number of siblings	2,945	4,856,912				
No sibling	1,088	1,710,472	35.2	33.2–37.3		
1–2 siblings	1,383	2,224,536	45.8	43.6–48.0		
3 and more siblings	474	921,904	19.0	17.2–20.9		
Food security	2,889	4,759,195				
Household food secure	1,721	2,774,220	58.3	56.1–60.5		
Household food insecure	514	867,222	18.2	16.5–20.0		
Individual food insecure	189	299,054	6.3	5.3–7.5		
Child hunger	465	818,699	17.2	15.6–19.0		

95% CI, 95% Confidence Interval; MYR, Malaysian Ringgit; 1 US Dollar = MYR 4.13 (as of November 14, 2025); SE, Standard Error.

a Source: Household Income and Basic Amenities Survey Report 2019, Department of Statistics Malaysia (66).

b Source: Household Expenditure Survey Report 2019, Department of Statistics Malaysia (22).

Children’s diet quality, evaluated using NAR and MAR across 15 nutrients, is presented in Table 2. Macronutrient intakes among children were highly adequate, with protein, carbohydrate, and fat achieving high NAR values of 0.99, 0.97, and 0.98, respectively. However, calcium (0.69), vitamin D (0.50) and potassium (0.31) adequacies were considerably lower compared with other micronutrients. The overall MAR was 0.84 among Malaysian children. To assess the nutritional status of children, BAZ, HAZ, and WAZ scores were calculated, as illustrated in Table 3. The prevalences of undernutrition, indicated by thinness/wasting, stunting and underweight, were 6.7, 8.9 and 10.4%, respectively.

**Table 2:** Nutrient intakes and adequacy level among children (*n* = 2,602).

Characteristics	Mean	SE	95%CI
Nutrient intake			
Energy (kcal)	1,388	12	1,365–1,412
Protein (g)	50.95	0.54	49.90–52.00
Carbohydrate (g)	188.2	1.7	184.8–191.6
Fat (g)	47.91	0.49	46.94–48.87
Vitamin A (mcg RE)	710.6	9.7	691.6–729.6
Vitamin D (mcg)	5.73	0.11	5.50–5.95
Vitamin C (mg)	77.89	1.80	74.37–81.42
Thiamine (mg)	1.06	0.01	1.03–1.09
Riboflavin (mg)	1.38	0.02	1.35–1.41
Cobalamin (mg)	3.03	0.07	2.90–3.16
Calcium (mg)	629.2	8.6	612.4–645.9
Phosphorus (mg)	704.2	8.0	688.6–719.8
Iron (mg)	11.67	0.15	11.37–11.96
Sodium (mg)	1,713	24	1,664-1,761
Potassium (mg)	1,088	11	1,065-1,111
NAR			
Energy	0.87	0	0.86–0.88
Protein	0.99	0	0.99–0.99
Carbohydrate	0.97	0	0.97–0.97
Fat	0.98	0	0.97–0.98
Vitamin A	0.94	0	0.94–0.95
Vitamin D	0.50	0.01	0.49–0.52
Vitamin C	0.87	0.01	0.86–0.89
Thiamine	0.92	0	0.91–0.93
Riboflavin	0.97	0	0.96–0.97
Cobalamin	0.79	0.01	0.77–0.80
Calcium	0.69	0.01	0.68–0.71
Phosphorus	0.86	0.01	0.85–0.87
Iron	0.97	0	0.96–0.97
Sodium	0.90	0	0.89–0.91
Potassium	0.31	0	0.31–0.32
MAR	0.84	0	0.83–0.84

95% CI, 95% Confidence Interval; MAR, Mean Adequacy Ratio; NAR, Nutrient Adequacy Ratio; SE, Standard Error.

**Table 3:** Nutritional status among Malaysian children (*n* = 2,973).

	Unweighted count (*n*)	Estimated population	Percentage (%)	95% CI	Mean	SE
Body weight status (BAZ)					−0.09	0.03
Thinness/wasting	191	329,976	6.7	5.7–7.9		
Normal	2,205	3,693,576	75.3	73.4–77.1		
Overweight	299	450,905	9.2	8.0–10.5		
Obese	278	429,822	8.8	7.7–10.0		
Height/length status (HAZ)					−0.58	0.02
Stunted	231	437,091	8.9	7.7–10.3		
Normal	2,742	4,467,188	91.1	89.7–92.3		
Underweight Status (WAZ) (0.50–9.99 years)	2,274	3,756,279			−0.51	0.03
Underweight	202	389,019	10.4	8.9–12.1		
Normal	2,072	3,367,260	89.6	87.9–91.1		

95% CI, 95% Confidence Interval; SE, Standard Error.

Table 4 shows food security characteristics in relation to child hunger by sociodemographic status. The prevalence of child hunger was higher in the East Coast (20.3%) and Southern (20.2%) regions (*p* < 0.01), as well as in rural areas (23.3%) (*p* < 0.001). Malay (19.4%) and Indian (19.6%) households had significantly higher prevalence of child hunger than Chinese households (6.5%) (*p* < 0.001). Most children who experienced child hunger came from households with low parental education (without formal education/primary school: 40.2% for mothers; 26.0% for fathers) (*p* < 0.001), unemployed mothers (26.3%), low monthly income (low income: 24.6%), low food expenditure (< MYR 783: 24.0%), and with three or more siblings (28.2%) (*p* < 0.001).

**Table 4:** Food secure characteristics by sociodemographic status (*n* = 2,889).

Characteristics	Non-child hunger (*n* = 2,424)		Child hunger (*n* = 465)		Chi-square value	*p*-value
	%	95%CI	%	95%CI		
Age group					1.331	0.849
0.5–0.9 years	85.7	72.0–93.3	14.3	6.7–28.0		
1.0–3.9 years	83.5	79.5–86.7	16.5	13.3–20.5		
4.0–6.9 years	83.0	79.9–85.7	17.0	14.3–20.1		
7.0–12.9 years	82.1	79.4–84.6	17.9	15.4–20.5		
Sex					0.293	0.658
Boys	83.2	80.6–85.5	16.8	14.5–19.4		
Girls	82.4	80.0–84.6	17.6	15.4–20.0		
Residence					27.171	<0.001
Rural	76.7	73.1–80.1	23.3	19.9–26.9		
Urban	85.0	83.0–86.8	15.0	13.2–17.0		
Ethnicity					44.983	<0.001
Malay	80.6	78.4–82.6	19.4	17.4–21.6		
Chinese	93.5	91.5–95.0	6.5	5.0–8.5		
Indian	80.4	74.3–85.4	19.6	14.6–25.7		
Others	85.1	68.1–93.9	14.9	6.1–31.9		
Region					24.525	0.001
Central	88.0	84.3–90.8	12.0	9.2–15.7		
East coast	79.7	76.1–83.0	20.3	17.0–23.9		
Northern	82.8	79.5–85.8	17.2	14.2–20.5		
Southern	79.8	76.1–83.1	20.2	16.9–23.9		
Paternal education					115.318	<0.001
Non-schooling/primary school	74.0	63.8–82.1	26.0	17.9–36.2		
Secondary school	77.0	74.3–79.6	23.0	20.4–25.7		
Tertiary school	92.2	90.1–93.8	7.8	6.2–9.9		
Maternal education					194.034	<0.001
Non-schooling/primary school	59.8	47.8–70.7	40.2	29.3–52.2		
Secondary school	74.7	71.7–77.4	25.3	22.6–28.3		
Tertiary school	92.5	90.6–94.0	7.5	6.0–9.4		
Maternal employment status					107.502	<0.001
Not working	73.7	70.3–76.8	26.3	23.2–29.7		
Working	88.7	86.8–90.4	11.3	9.6–13.2		
Total monthly household income					164.472	<0.001
B40 (< MYR 4850)	75.4	72.8–77.8	24.6	22.2–27.2		
M40 (MYR 4850–10,959)	93.6	91.2–95.3	6.4	4.7–8.8		
T20 (> MYR 10959)	95.0	90.2–97.5	5.0	2.5–9.8		
Monthly household food expenditure					81.087	<0.001
< MYR 783	76.0	73.0–78.8	24.0	21.2–27.0		
≥MYR 783	88.8	86.8–90.6	11.2	9.4–13.2		
Number of siblings					64.091	<0.001
No siblings	87.3	84.6–89.6	12.7	10.4–15.4		
1–2 siblings	84.5	82.0–86.8	15.5	13.2–18.0		
3 or more siblings	71.8	66.8–76.4	28.2	23.6–33.2		

95% CI, 95% Confidence Interval; MYR, Malaysian Ringgit; 1 US Dollar = MYR 4.13 (as of November 14, 2025). Significant differences were determined using complex samples Pearson Chi-square test.

In terms of diet quality, boys (0.84) exhibited slightly higher MAR than girls (0.83) on average (*p* < 0.01), with significant differences found in urban population (0.84) compared to their rural counterparts (0.82) (*p* < 0.01) (Table 5). Although these differences were statistically significant, the absolute differences were small. Children with working mothers (0.85) had higher MAR than those with non-working mothers (0.82) (*p* < 0.001). Higher MAR were also observed among children with younger age (0.5–0.9 years: 0.89; 1.0–3.9 years: 0.90), from Chinese or other ethnicities, households with tertiary educated mothers, and those with fewer siblings.

**Table 5:** Mean adequacy ratio by socioeconomic status (*n* = 2,602).

Characteristics	MAR
	Mean (95%CI)
Age group	
0.5–0.9 years	0.89 (0.86–0.92)^bc^
1.0–3.9 years	0.90 (0.89–0.91)^c^
4.0–6.9 years	0.86 (0.86–0.87)^b^
7.0–12.9 years	0.79 (0.78–0.80)^a^
Sex	
Boys	0.84 (0.84–0.85)**
Girls	0.83 (0.82–0.83)
Residence	
Rural	0.82 (0.81–0.83)
Urban	0.84 (0.83–0.85)**
Ethnicity	
Malay	0.83 (0.83–0.84)^a^
Chinese	0.85 (0.84–0.86)^b^
Indian	0.83 (0.81–0.85)^a^
Others	0.87 (0.83–0.90)^b^
Region	
Central	0.84 (0.83–0.85)^ab^
East coast	0.82 (0.81–0.83)^a^
Northern	0.84 (0.83–0.85)^ab^
Southern	0.85 (0.84–0.86)^b^
Paternal Education	
Non-schooling/primary school	0.82 (0.80–0.85)
Secondary school	0.83 (0.83–0.84)
Tertiary school	0.84 (0.83–0.85)
Maternal education	
Non-schooling/primary school	0.83 (0.79–0.86)^ab^
Secondary school	0.83 (0.82–0.83)^a^
Tertiary school	0.85 (0.84–0.85)^b^
Maternal employment status	
Not working	0.82 (0.81–0.83)
Working	0.85 (0.84–0.85)***
Total monthly household income	
B40 (< MYR 4850)	0.83 (0.82–0.84)
M40 (MYR 4850–10,959)	0.84 (0.84–0.85)
T20 (> MYR 10959)	0.84 (0.82–0.85)
Monthly household food expenditure	
< MYR 783	0.84 (0.83–0.85)
≥MYR 783	0.83 (0.83–0.84)
Number of siblings	
No siblings	0.85 (0.84–0.85)^b^
1–2 siblings	0.83 (0.83–0.84)^a^
3 and more siblings	0.82 (0.81–0.84)^a^

MAR, Mean Adequacy Ratio; 95% CI, 95% Confidence Interval; MYR, Malaysian Ringgit; 1 US Dollar = MYR 4.13 (as of November 14, 2025). Significant differences within socioeconomic status were determined using complex samples ANOVA were indicated using alphabet or **p < 0.01, and ***p < 0.001.

The growth indicators of children by sociodemographic status are detailed in Table 6. Children from younger age groups, households with lower income and lower food expenditure had significantly lower HAZ, WAZ, and BAZ scores (*p* < 0.05). Besides, BAZ was significantly lower among girls than boys (*p* < 0.01), while children from rural areas had lower HAZ scores (*p* < 0.01). However, Malay children, children with unemployed mothers, and households with a higher number of siblings exhibited significantly lower HAZ and WAZ scores (*p* < 0.05).

**Table 6:** Growth indicators of children by socioeconomic status.

Characteristics	BAZ	HAZ	WAZ
	Mean (95%CI)	Mean (95%CI)	Mean (95%CI)
Age group			
0.5–0.9 years	−0.66 (−0.93–(−0.38))^a^	−0.58 (−0.92–(−0.23))^abc^	−0.82 (−1.14–(−0.49))^ab^
1.0–3.9 years	−0.23 (−0.34–(−0.13))^b^	−0.88 (−0.98–(−0.77))^a^	−0.71 (−0.82–(−0.59))^a^
4.0–6.9 years	−0.23 (−0.33–(−0.12))^b^	−0.66 (−0.74–(−0.58))^b^	−0.55 (−0.65–(−0.45))^b^
7.0–12.9 years	0.09 (−0.01–0.20)^c^	−0.40 (−0.46–(−0.33))^c^	−0.24 (−0.37–(−0.11))^c^
Sex			
Boys	−0.01 (−0.10–0.08)**	−0.58 (−0.65–(−0.52))	−0.48 (−0.57–(−0.39))
Girls	−0.18 (−0.26–(−0.10))	−0.59 (−0.65–(−0.52))	−0.55 (−0.64–(−0.46))
Residence			
Rural	−0.04 (−0.16–0.08)	−0.70 (−0.79–(−0.62))	−0.58 (−0.70–(−0.46))
Urban	−0.11 (−0.18–(−0.04))	−0.54 (−0.60–(−0.48))**	−0.49 (−0.57–(−0.41))
Ethnicity			
Malay	−0.11 (−0.19–(−0.04))	−0.69 (−0.75–(−0.63))^a^	−0.61 (−0.69–(−0.53))^a^
Chinese	−0.10 (−0.19–0)	−0.32 (−0.40–(−0.25))^b^	−0.35 (−0.44–(−0.26))^b^
Indian	0.05 (−0.19–0.29)	0.01 (−0.14–0.15)^c^	0.14 (−0.10–0.39)^c^
Others	0.06 (−0.36–0.47)	−0.53 (−0.89–(−0.16))^ab^	−0.31 (−0.76–0.13)^abc^
Region			
Central	−0.26 (−0.38–(−0.13))^a^	−0.53 (−0.64–(−0.43))^bc^	−0.62 (−0.75–(−0.48))^a^
East coast	−0.09 (−0.21–0.02)^ab^	−0.71 (−0.80–(−0.62))^a^	−0.64 (−0.75–(−0.52))^a^
Northern	−0.01 (−0.13–0.12)^b^	−0.46 (−0.55–(−0.37))^c^	−0.37 (−0.49–(−0.24))^b^
Southern	0.04 (−0.08–0.15)^b^	−0.62 (−0.71–(−0.53))^ab^	−0.39 (−0.51–(−0.27))^b^
Paternal education			
Non–schooling/primary school	−0.19 (−0.46–0.07)	−0.65 (−0.89–(−0.42))	−0.65 (−0.96–(−0.34))
Secondary school	−0.08 (−0.17–0.01)	−0.58 (−0.64–(−0.51))	−0.46 (−0.55–(−0.37))
Tertiary school	−0.10 (−0.20–(−0.01))	−0.58 (−0.65–(−0.50))	−0.55 (−0.65–(−0.44))
Maternal education			
Non–schooling/primary school	−0.13 (−0.43–0.16)	−0.52 (−0.81–(−0.24))	−0.43 (−0.73–(−0.12))
Secondary school	−0.12 (−0.22–(−0.03))	−0.63 (−0.70–(−0.56))	−0.55 (−0.65–(−0.46))
Tertiary school	−0.06 (−0.15–0.02)	−0.55 (−0.62–(−0.48))	−0.49 (−0.58–(−0.40))
Maternal employment status			
Not working	−0.17 (−0.27–(−0.06))	−0.68 (−0.76–(−0.60))	−0.66 (−0.77–(−0.55))
Working	−0.05 (−0.12–0.03)	−0.52 (−0.58–(−0.46))**	−0.43 (−0.51–(−0.35))**
Total monthly household income			
B40 (< MYR 4850)	−0.17 (−0.25–(−0.09))^a^	−0.65 (0.03–(−0.59))^a^	−0.60 (−0.68–(−0.51))^a^
M40 (MYR 4850–10,959)	−0.02 (−0.13–0.08)^b^	−0.52 (0.04–(−0.44))^b^	−0.40 (−0.50–(−0.29))^b^
T20 (> MYR 10959)	0.10 (−0.12–0.33)^b^	−0.37 (0.08–(−0.22))^b^	−0.39 (−0.63–(−0.14))^ab^
Monthly household food expenditure			
< MYR 783	−0.18 (−0.27–(−0.09))	−0.71 (−0.79–(−0.64))	−0.62 (−0.71–(−0.52))
≥MYR 783	−0.02 (−0.11–0.06)**	−0.46 (−0.53–(−0.40))***	−0.42 (−0.51–(−0.33))**
Number of siblings			
No siblings	−0.04 (−0.14–0.06)	−0.45 (−0.52–(−0.37))^b^	−0.36 (−0.47–(−0.27))^c^
1–2 siblings	−0.13 (−0.22–(−0.04))	−0.65 (−0.72–(−0.58))^a^	−0.55 (−0.64–(−0.45))^b^
3 and more siblings	−0.15 (−0.30–0.01)	−0.72 (−0.84–(−0.59))^a^	−0.75 (−0.91–(−0.59))^a^

95% CI, 95% Confidence Interval; MYR, Malaysian Ringgit; 1 US Dollar = MYR 4.13 (as of November 14, 2025). Significant differences within socioeconomic status were determined using Complex Samples ANOVA were indicated using alphabet or **p < 0.01, and ***p < 0.001.

## 4 Discussion

The present study highlights the significant impact of socioeconomic disparities on food security, diet quality, and growth outcomes among Malaysian children aged 0.5 to 12.9 years. Despite one-fifth of the children suffering from hunger, their overall diet quality was moderate. While basic macronutrients requirements were generally met, deficiencies persisted in essential micronutrients, particularly calcium, vitamin D, and potassium, indicating a form of “hidden hunger” commonly seen in resource-limited settings. The findings of the present study also underscore nutritional inequities, where children from higher socioeconomic backgrounds, especially those living in urban areas and with working mothers, had better diet quality. The anthropometric findings further reflect the influence of social determinants, as children from low-income, rural households, and those whose parents had lower education level were disproportionately more likely to have suboptimal growth indicators.

Children residing in rural areas were more likely to experience hunger, and had poorer diet quality and lower HAZ scores, indicating a greater risk of stunting. In line with our study, Naser et al. (34) pointed out the differences between urban and rural areas in north-eastern region of Peninsular Malaysia, with children in rural areas being more affected by food insecurity and hunger than their urban peers, despite poverty in both settings. Another recent evidence from Malaysia also suggests that children in rural areas are 1.35 times more likely to experience undernutrition compared to their urban peers (18). These disparities are due to the availability and accessibility of food, lower parental awareness of nutrition, and inadequate health infrastructure in rural areas (35). Therefore, children living in rural areas are less likely to meet food group recommendations, and tend to have less diverse and less nutrient-rich diets, leading to dependence on calorie-dense staples (36).

Parental education, particularly maternal education level, is crucial to prevent children from hunger. Besides, mothers with higher educational attainment were associated with better diet quality among children. Educated parents tend to have better nutrition knowledge, employment opportunities, and decision-making capacity regarding food purchases and feeding practices (37, 38). These advantages contribute to more balanced diets, optimal child feeding practices, improved healthcare utilisation, all of which reduce malnutrition risks and support children’s well-being (39). Maternal employment demonstrated a dual role in shaping children’s nutrition. In many cases, particularly in urban settings, working mothers contribute to greater household finances and food affordability, which can improve dietary diversity and quality (40). Our findings support this, showing that children of working mothers generally experienced less hunger and had better diet quality. Studies from Ethiopia and China similarly reported lower rates of undernutrition (41) and healthier BMIs (42) among children with employed mothers, emphasising the importance of maternal income for children’s nutrition.

Having non-working mothers and larger family sizes were associated with poorer nutritional outcomes, especially lower HAZ and WAZ scores. This is due to the resource dilution, where limited food resources are shared among multiple children, reducing the opportunity for each child to receive adequate food and nutrient intake (43). In such households, children are more likely to experience hunger, have a less varied diet, and suffer from undernutrition. The burden of non-working mothers in large households may limit the ability to ensure optimal feeding and hygiene practices, further compromising child health (44). Nonetheless, maternal employment may also pose time-related challenges. A study in urban settings have shown that working mothers often face time constraints (45). These constraints are associated with greater reliance on processed and convenience foods, leading to lower diet quality among children (46). With the rising prevalence of dual-income families in many societies, including Malaysia, supportive policies such as flexible work schedules, affordable childcare, and community meal program, are increasingly important to ease time burdens and promote healthier eating practices among children.

Household income also remains a key factor influencing food security and growth. Children from poorer families were more vulnerable to food insecurity and hunger, in accordance with national findings that linked poverty to diminished food access and reduced quantity of food intake (47). A local study conducted in Kuala Lumpur, a metropolitan area, revealed that 52% of children from low-income families consumed fewer than three meals daily (48). Low income households often experience financial constraints that limit food expenditure (49), and usually rely on low-cost, calorie-dense, and nutrient-poor foods, leading to limited dietary diversity and insufficient nutrient intake among children (50). This restricts the intake of key micronutrients, which are essential for growth and development (51), such as calcium, vitamin D, and potassium. The downstream effect of such poor diet quality is potentially reflected in children’s lower HAZ, WAZ, and BAZ scores.

In our study, diet quality appeared to decline with age, especially among older children. In support of this trend, Koo et al. (52) found that fewer children aged 10 to 12 years met the recommendations for nutrient-rich food groups, such as cereals, grains, meat, poultry, legumes, and dairy, compared to their younger peers aged 7 to 9 years. This decline may be partly attributed to changes in parenting styles, as older children experience less parental supervision (53). Younger children benefit from authoritative parenting, characterised by role modeling of healthier diets and structured mealtime routines, linked to improved diet quality. Conversely, older children growing under authoritarian or permissive parenting combined with poor communication, may be more easily influenced by peer norms and an unhealthy food environment (54). Although diet quality decreases with age, our findings show that younger children, particularly children under the age of five, have significantly lower HAZ, WAZ, and BAZ scores. Younger children undergo a rapid growth phase that demands consistent intake of nutrient-dense foods, and are vulnerable to short-term dietary inadequacy and irreversible growth faltering in this critical period (55). Besides, delay in child growth typically begins after six months of age, coinciding with the introduction of complementary feeding, which may not always meet nutritional requirements (56). Thus, poorer growth outcomes are observed in younger children. However, declining diet quality in older children remains a concern, as poor dietary intakes during late childhood and preadolescence may adversely affect pubertal development. Studies suggest that better diet quality during childhood is tied to delayed puberty onset, suggesting potential long-term health benefits (57, 58).

Interestingly, sex differences were observed in both diet quality and growth outcomes. Although boys demonstrated slightly better diet quality than girls, the magnitude of the difference was minimal, and its clinical relevance is likely limited. However, the consistent direction of findings may be attributed to their tendency to consume larger portion sizes (59). These larger portions can lead to proportionately higher nutrient intake, potentially contributing to improved diet adequacy. Girls, in contrast showed lower BAZ scores, which potentially reflects unmet calorie and nutrient requirements that hinder optimal growth and development (60). These patterns indicate that even small gaps in adequacy, if persistent, could accumulate and lead to disparities in long-term growth outcomes. In addition, societal and cultural perceptions of body image may further influence girls’ dietary behaviors. Concerns about appearance or pressure to maintain a certain body shape can lead to restrictive eating patterns, reduced food intake and increased vulnerability to undernutrition (61).

Ethnic background also plays a significant role in shaping child nutrition outcomes. Malay and Indian children tend to suffer from hunger, likely due to socioeconomic disadvantages such as lower household income and larger family size (62). Chinese children had better diet quality, which aligns with findings showing that their dietary habits were less likely to follow discretionary patterns such as Western food and sugary beverages. This reduced adherence to unhealthy patterns may provide greater opportunities to consume more nutritious food options (63). These distinctions in food selection may explain the observed differences in diet quality among ethnic groups. Furthermore, these nutritional advantages were reflected in better growth outcomes, with Malay and Indian children showing lower HAZ and WAZ scores, suggesting a cumulative impact of child hunger and suboptimal diets on physical development.

This study provides comprehensive insights into the nutritional status and growth patterns of Malaysian children aged 0.5 to 12.9 years, with particular focus on their association with socioeconomic and household factors. A key strength lies in the use of a nationally representative and demographically diverse sample of Malaysian children across age groups, ethnicities, geographic location, and income levels. This enhances the external validity of the findings and ensures their relevance for public health and policy planning. The integration of multiple anthropometric indicators with detailed macro- and micronutrient intake assessments offers a nuanced understanding of the interplay between diet quality and child growth. Furthermore, stratified analyses by household composition, including maternal employment and number of siblings, add critical contextual depth. This reveals the multidimensional impact of social determinants on child nutrition. The alignment of findings with national dietary surveys, while also uncovering unique household-level disparities, underscores the study’s contribution to evidence-based policymaking in Malaysia.

Nevertheless, several limitations should be acknowledged. Dietary assessments based on 24-h recall and the utilisation of questionnaires are inherently subject to recall bias and social desirability bias. Although standardised protocols with probing techniques were employed to minimise these biases, residual misreporting may persist. Moreover, this study does not account for other probable confounders, such as physical activity, psychosocial stressors, or access to healthcare, all of which may influence nutritional status and growth. While the study includes diverse participants, it was conducted only in Peninsular Malaysia due to COVID-19 movement restrictions, which may have led to under-representation of certain subpopulations or remote rural areas, limiting the overall generalisability of the findings to all Malaysian children. Future longitudinal studies incorporating these additional dimensions are warranted to more accurately determine the causal pathways and inform targeted, equitable nutrition interventions.

## 5 Conclusion

This study confirms the association of socioeconomic disparities with compromised food security, poorer diet quality, and suboptimal growth outcomes among Malaysian children. Despite an overall diet quality assessed as moderate, children continue to experience critical micronutrients inadequacies and poor growth outcomes. These vulnerabilities remain pronounced among children from low-income households, rural areas, families with lower parental education, non-working mothers, and larger households. Variations by age, sex, ethnicity, and geographic location further reflect the multifaceted nature of malnutrition. Addressing both economic and educational inequalities within households is essential for improving child nutrition in Malaysia. Targeted, equity-oriented interventions should prioritise nutrition education and social support for parents, particularly mothers, alongside measures that improve the affordability and accessibility of nutrient-dense foods. Community- and school-based programmes, alongside policies that strengthen household food security, enhance maternal nutrition knowledge, and promote healthy child-feeding practices, can help bridge dietary gaps and support optimal growth among vulnerable children. Our findings highlight the interconnectedness of child nutrition, household income status, and parental education, all of which impact immediate health outcomes and may exacerbate lasting effects on workforce competitiveness of future generations. The findings directly support the United Nations’ Sustainable Development Goals (SDG) 2 (Zero Hunger) and SDG 3 (Good Health and Well-being) (64) by tackling food insecurity and malnutrition while contributing to SDG 10 (Reduced Inequalities) through strategies aimed at narrowing socioeconomic gaps. Furthermore, these insights reinforce Malaysia’s National Plan of Action for Nutrition (NPAN) (65), which prioritises equitable interventions to improve diet quality, growth outcomes and overall well-being of children.

## Data Availability

The raw data supporting the conclusions of this article will be made available by the authors, without undue reservation.

## Group members of SEANUTS II Malaysia study group

The SEANUTS II Malaysia Study Group comprises the following. Universiti Kebangsaan Malaysia: Bee Koon Poh, Jyh Eiin Wong, Nik Shanita Safii, Nor MF Farah, Nor Aini Jamil, Razinah Sharif, Caryn Mei Hsien Chan, Swee Fong Tang, Lei Hum Wee, Siti Balkis Budin, Denise Koh, Abd. Talib Ruzita, Nur Zakiah Mohd Saat, Mohd Jamil Sameeha, A. Karim Norimah, See Meng Lim, Jasmine Siew Min Chia, Shoo Thien Lee. FrieslandCampina: Ilse Khouw, Swee Ai Ng, Nanda de Groot, Cécile M. Singh-Povel, Jan M.W. Geurts.
